# A possible model for estimating birth length of babies from common parental variables using a sample of families in Lagos, Nigeria

**DOI:** 10.4314/ahs.v21i1.44

**Published:** 2021-03

**Authors:** Idowu Adewumi Taiwo, Adenike Adeleye, Ijeoma Chinwe Uzoma

**Affiliations:** 1 Genetics Unit; Department of Cell Biology and Genetics, Faculty of Science, University of Lagos, Lagos, Nigeria; 2 Molecular-Haematology Laboratory; Department of Medical Laboratory Science, Faculty of Health Sciences and Technology, College of Medicine, University of Nigeria Nsukka, Enugu Campus, Enugu, Nigeria

**Keywords:** Parental anthropometrics, birth length, association, model, correlation

## Abstract

**Background:**

Length at birth is important for evaluating childhood growth and development. It is of interest in Pediatrics because of its implications for perinatal and postnatal morbidity and mortality. Predicting birth length will be useful in anticipating and managing possible complications associated with pregnancy and birth of babies with abnormal birth length.

**Objective:**

The aim was to identify easily accessible parental determinants of baby's birth length in Lagos, Nigeria, using a sample of patients attending a government hospital.

**Methods:**

Parental anthropometrics and other data were obtained from 250 couples by actual measurements, oral interviews and questionnaires. Baby's birth length was measured immediately after delivery by qualified, a well-trained obstetric nurse, and association between parental and offspring parameters were assessed.

**Results:**

Weight gain, maternal weight, parity and mid-parental height were the significant parental explanatory variables of offspring birth length. They were the most suitable variables for a generated model for predicting babies' birth length from parental variables in the study.

**Conclusion:**

A model that might be useful for predicting babies' birth length from easily accessible parental variables was produced. This model may complement ultrasonographic data for predicting baby's birth length with a view to achieving better perinatal and postnatal care.

## Introduction

Birth length is an important neonatal measurement taken at birth not only because of its association with adult height but also because of its importance in clinical medicine and quantitative genetics. Considerable attention had been focused on birth weight because of the general belief that it is the best indicator of foetal growth and birth size when compared to other neonatal parameters. Furthermore, birth weight has been identified by several researchers as one of the most important factors of quality of life in adulthood according to the fetal origin hypothesis proposed by Baker^1^. This is consistent with the view of Lubinsky in a recent review, which includes the role of epigenetic modification in fetal programming^2^. However, Sorensen et al.^3^ and, more recently, Silva et al.^4^ suggested that birth length, when compared to birth weight, may be a better indicator of foetal growth and birth size because length at birth is less influenced by nutritional factors and placental function. Thus, there seems to be greater biological protection for foetal length changes than for foetal weight changes^5^.

The association between birth length and several perinatal and postnatal morbidity and mortality has been the subject of many studies. There is a strong association between birth length and late onset wheeze in children of between 2 months to 4 years old^6^. Yuan et al.^7^ also reported that low birth length correlated with risk of hospitalization due to infections during childhood. Vos et al.^8^ found that birth length was associated with coronary heart disease in women while Silva *et al.*^4^ and Maehle et al.^9^ observed a relationship between birth length and breast cancer.

In view of the high level of poverty, illiteracy and ignorance especially in low income countries, many expectant mothers in these regions do not attend antenatal clinics; foetal growth including foetal length could therefore not be properly monitored. By the time pregnancy is at full term and parturition is about to commence, it might be rather late to take significantly effective steps to prevent problems associated with abnormal birth length. Thus, ability to predict abnormal birth length from parental anthropometrics may enhance identification of parents at risk of having babies with abnormal birth length to enable proper education and early surveillance. This, hopefully, would make early preventive strategies possible.

A good number of studies have been carried out on heritability and parental contribution to birth length in Caucasian and Asian populations, but little or no similar studies exist in Nigeria and many other low income countries of Africa. Since heritability is a population-specific attribute, the need to carry out similar studies in Nigeria cannot be overemphasized. The present study was therefore undertaken to see the possibility of developing a model for predicting offspring's birth length from simple parental variables using a sample of patients attending a government hospital in Lagos.

Although assessing baby's development through ultra-scan technique is not new, its use in estimating neonatal anthropometric parameters has not being totally efficient^10^. The use of ultrasound is partially dependent on the date of last menstruation; however, menstruation may be irregular, and many mothers may not remember the exact date when menstruation commenced. Moreover, ultrasound scan is not widely available in low income countries, especially in the rural settings, where most pregnant mothers attend traditional birth centers and spiritual homes. Predicting baby's birth length using parental anthropometric attributes as suggested by this study might not only help in identifying families at risk (for surveillance purposes), but also complement the already existing methods (e.g. ultrascan) for better and more accurate prediction of birth length.

## Methods

### Subjects and exclusion criteria

Two hundred and fifty couples attending the ante-natal clinic of General Hospital, Surulere, Lagos, Nigeria, were recruited for the study after successfully seeking approval from Institution's Ethical Committee. The major inclusion criterion was singleton pregnancy while the criteria for exclusion included presence of diabetes, hypertension, malnutrition, anaemia, malignancies and HIV/AIDS. Other exclusion criteria were presence of complications such as pre-eclampsia/eclampsia, antepartum hemorrhage, uterine fibroid or any other uterine or placental abnormalities. These were verified from medical records. Sufficiency of the sample size (250 family units) for the study was determined using the formula below.^11^

n [(Z_α/2_)^2^ P(1-P) ] / E^2^.......................(1)

Thus, given a population proportion (P) of 0.5 with a margin of error (E) of 0.07 at 95% confidence level i.e Zα/2 = 1.96, the appropriate sample size (n) was found to be 196 couples (taking a couple as a unit). In view of this, the sample size of 250 couples used for this study was considered adequate.

### Questionnaires and their administration

After explanation of the purpose and procedure to the study participants, they were requested to fill consent forms and questionnaires administered by the researchers to obtain personal data including gender, ethnicity, weight before pregnancy (pre-pregnancy weight) parity, gravidity, and age. The participants were guided in filling the questionnaire where necessary. Information about parental anthropometrics like mothers and fathers' weights, heights were obtained through actual measurements by a trained nurse, who recorded the measurements under the section of the questionnaire tagged “For Official Use Only”.

In order to get the combined effect of the height of both parents on their babies' birth length and birth weight, mid-parental height and weight were derived according to the formulas:

Mid-parental height = father's height + mother's height)/2 ...………… (2)

Mid-parental weight = father's weight + mother's weight)/2 ...…..……. (3)

Mid-parental BMI = father's BMI + mother's BMI)/2 ...…………...….…. (4)

### Measurement of offspring parameters

Immediately after delivery, the baby was cleaned of blood and other post-delivery fluids. The sex of the baby was determined and those with ambiguous genitalia were excluded from the study. Measurement of birth length and other neonatal parameters routinely measured after delivery e.g. birth weight, and head circumference) was completed within 30 minutes of delivery by a trained obstetric nurse. However, since birth length was the only baby's parameter of interest in this study, it was the only neonatal variable involved in the study. Thus, two nurses were involved in the study: a general nurse who took the parental anthropometrics and an obstetric nurse who took the neonatal measurements.

### Data analysis

The raw data were analyzed statistically using Microsoft Excel (2007) and IBM Statistics / Statistical Package for Social Scientists (SPSS) Statistics (Version 25) software packages. The initial analysis was to carry out descriptive statistics followed by comparison of mean± SE by the Student's t-test. Simple correlation procedure was used to generate a pairwise correlation matrix which was followed by partial correlation analysis to see whether associations seen with simple correlation analysis were influenced by intercorrelations. Based on this, dimension reduction using Principal Component Analysis (PCA) was used to remove redundant highly correlated variables to produce smaller number of uncorrelated variables which could effectively explain and predict baby's birth length. We used multiple regression analysis to generate regression models which were subjected to analysis of variance (ANOVA) for test of goodness of fit. In all cases, P<0.05 was considered significant.

## Results

Summary statistics of the parents is given in Table 1. Fathers were generally taller than mothers in view of the mean± SE height of fathers which was 173.9± 0.51 cm as compared to the height of mothers (168.1± 0.57 cm; P<0.001). Fathers also had higher body weight because their body mass of 78.3± 0.44 kg was significantly higher (P<0.001) than that of the mothers (68.1± 0.55 kg). The maternal and paternal BMI were 24.3 ± 0.24 kg/m2 and 25.9± 0.17 kg/m^2^ respectively. Parity shows the highest variation (51.8) while paternal height showed the least variation (coefficient of variation = 4.6).

**Table 1:** Descriptive statistics of parents

	Min.	Max.	Mean± SE	Std. Dev.	Coef. of Var. (%)
**Maternal Age** **(yrs.)**	18	42	30.2±0.33	5.2	17.2
**Maternal Weight** **Gain (kg)**	3	19	10.9±0.15	2.4	22.1
**Paternal** **Weight (kg)**	60	101	78.3±0.44	6.9	8.8
**Maternal Weight** **(Kg)**	42	90	68.1±0.55	8.6	12.6
**Parity**	1	4	1.6±0.05	0.8	51.8
**Paternal Height** **(cm)**	150	196	173.9±0.51	8.0	4.6
**Maternal Height** **(cm)**	139	198	168.1±0.57	8.9	5.3
**Paternal** **BMI (kg/m^2^)**	14.5	35.1	25.9±0.17	2.7	10.3
**Maternal BMI (kg/m^2^)**	14.8	46.6	24.3±0.24	3.8	15.7
**Mid Parental** **Weight (kg)**	57	89	73.2±0.41	6.4	8.7
**Mid Parental** **Height (kg)**	103	197	170.6±0.59	9.2	5.4
**Mid Parental** **BMI (kg/m^2^)**	16.6	37	25.1±0.18	2.8	11.0

**Figure 1:**
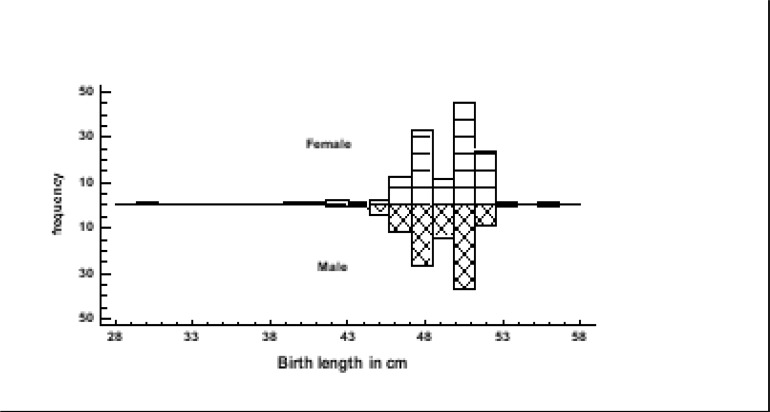
Birth length Distribution of male and female babies

In Table 2, the mean ± SE of birth length of female babies (49.0± 0.2) was not significantly different (P>0.05) from that of male babies (48.9± 0.2). Moreover, both male and female babies had similar distribution of birth length (Fig. 1). Both groups were therefore treated as a single group to increase the sample size and, therefore, the power of statistical analysis. The summary statistics of the babies is presented in Table 2.

**Table 2:** Comparative birth length of male and female babies showing no significant difference between means of male and female babies

	Baby's Gender	Mean	Std. Dev	Std. Error	Coeff of Var. (%)
**Birth Length** **(cm)**	Female	49.0	2.9	0.2	5.9
	Male	48.9	2.0	0.2	4.2
	
	**Combined**	**49.0**	**2.6**	**0.2**	**5.2**

t=0.29; P=0.78: no statistically significant difference between the birth length of male and female babies

Many of the studied variables were intercorrelated as shown in Table 3. Mid parental BMI and maternal BMI were the most correlated variables (r = 0.9, P<0.001). Other pairs of variables that are highly correlated included mid parental weight/maternal weight (r = 0.86, P<0.001), mid parental BMI/paternal BMI (r = 0.78, P <0.001), mid parental height/maternal height (r = 0.78, P<0.001). Mid parental height/maternal age, as well as maternal age/weight gain may be considered the most uncorrelated, as these pairs have the least correlation coefficient among the studied variables (r = 0.004, P=0.964) and (r = 0.004, P = 0.956) respectively.

**Table 3:** Correlation Matrix Showing Intercorrelations between the Studied Variables

	MAT AGE	WT GAIN	PAT WT	MAT WT	PAT HT	MAT HT	PAT BMI	MAT BMI	BIRTH LT	HEAD CIRC	MID PAR WT	MID PAR HT	MID PAR BMI	Parity
**MAT** **AGE**	1													
**WT** **GAIN**	0.00 (0.96)	1												
**PAT** **WT**	0.14* (0.03)	0.06 (0.37)	1											
**MAT** **WT**	0.31** (0.00)	-0.25** (0.00)	0.35** (0.00)	1										
**PAT** **HT**	0.01 (0.85)	0.01 (0.89)	0.46** (0.00)	0.07 (0.26)	1									
**MAT** **HT**	-0.03 (0.67)	-0.02 (0.72)	0.25** (0.00)	0.22** (0.00)	0.57** (0.00)	1								
**PAT** **BMI**	0.03 (0.63)	0.03 (0.67)	0.46** (0.00)	0.26** (0.00)	-0.51** (0.00)	-0.28** (0.00)	1							
**MAT** **BMI**	0.26** (0.00)	-0.20** (0.00)	0.11 (0.10)	0.68* (0.00)	-0.36** (0.00)	-0.53** (0.00)	0.43** (0.00)	1						
**BIRTH** **LT**	0.17** (0.01)	0.16* (0.01)	0.10 (0.11)	0.11 (0.09)	0.19** (0.01)	0.19** (0.01)	-0.11 (0.08)	-0.06 (0.33)	1					
**HEAD** **CIRC**	0.20** (0.00)	0.16* (0.01)	0.26** (0.00)	0.10 (0.12)	0.11 (0.09)	-0.03 (0.63)	0.12 (0.06)	0.09 (0.17)	0.41** (0.00)	1				
**MID** **PAR** **WT**	0.29** (0.00)	-0.13* (0.05)	0.77** (0.00)	0.86** (0.00)	0.29** (0.00)	0.28** (0.00)	0.42** (0.00)	0.51** (0.00)	0.13* (0.04)	0.22** (0.00)	1			
**MID** **PAR** **HT**	0.00 (0.95)	-0.06 (0.32)	0.27** (0.00)	0.15* (0.02)	0.70** (0.00)	0.78** (0.00)	-0.40** (0.00)	-0.43** (0.00)	0.16 (0.07)	-0.02 (0.71)	0.25** (0.00)	1		
**MID** **PAR** **BMI**	0.20** (0.00)	-0.13* (0.05)	0.30** (0.00)	0.60** (0.00)	-0.49** (0.00)	-0.50** (0.00)	0.78** (0.00)	0.90** (0.00)	-0.10 (0.13)	0.12 (0.06)	0.56** (0.00)	-0.49** (0.00)	1	
**Parity**	0.41 (0.00)	0.08 (0.22)	-0.01 (0.91)	0.00 (0.99)	0.11 (0.09)	-0.00 (0.98)	-0.09 (0.17)	0.01 (0.85)	0.15 (0.02)	0.10 (0.13)	0.01 (0.90)	0.08 (0.20)	-0.05 (0.44)	1

Note: P values are in parentheses. P<0.05–significant; P<0.01–very significant; P<0.001–highly significant. Key: MAT-Maternal age; PAT-Paternal; WT-Weight; HT-Height; LT-Length; MIDPAR-Midparental.

Many of the intercorrelations revealed by simple correlation analysis vanished with partial correlation (Table 4). In Table 4 for instance, the simple correlation between mid-parental BMI and maternal age was 0.2 (P<0.001), but it was not observed with partial correlation (r= 0.0002; P>0.05). Likewise, the correlation between mid-parental BMI and maternal height reduced from 0.5 (P<0.001) to 0.009 when partial correlation procedure was performed (P>0.05). The pattern of the results was similar for maternal BMI/maternal age, paternal BMI/paternal weight, and birth length/ paternal height. However, with some variables, intercorrelations observed with simple correlation remained after partial correlation analysis. This is exemplified by the high correlation between mid-parental BMI and maternal BMI which was 0.899 (P<0.001) with simple correlation and 0.998 (P< 0.001) with partial correlation. Similar pattern of result was obtained for correlation between mid-parental weight and paternal weight.

**Table 4:** Results of partial bivariate correlation controlling for other variables

	MAT AGE	WT GAIN	PAT WT	MAT WT	PARITY	PAT HT	MAT HT	PAT BMI	MAT BMI	BIRTHLT	HEAD CIRC	MID PAR WT	MID PARHT	MID PAR BMI
**MATAGE**	1													
**WTGAIN**	0.01 (0.85)	1												
**PATWT**	0.12 (0.06)	-0.03 (0.68)	1											
**MATWT**	0.07 (0.31)	-0.12 (0.08)	-0.68 (0.00)	1										
**PARITY**	0.43 (0.00)	0.05 (0.43)	-0.14 (0.03)	-0.07 (0.27)	1									
**PATHT**	-0.24 (0.00)	-0.04 (0.51)	0.39 (0.00)	-0.10 (0.12)	0.16 (0.01)	1								
**MATHT**	-0.06 (0.38)	0.03 (0.70)	-0.05 (0.43)	0.46 (0.00)	-0.05 (0.46)	0.10 (0.14)	1							
**PATBMI**	-0.01 (0.83)	0.01 (0.85)	0.00 (0.94)	-0.05 (0.45)	-0.11 (0.08)	-0.10 (0.11)	-0.00 (0.96)	1						
**MATBMI**	-0.00 (0.97)	0.01 (0.85)	-0.02 (0.78)	-0.02 (0.78)	-0.12 (0.06)	-0.06 (0.38)	-0.05 (0.41)	-1.00 (0.00)	1					
**BIRTHLT**	0.03 (0.69)	0.13 (0.05)	0.04 (0.58)	0.05 (0.43)	0.09 (0.15)	-0.01 (0.84)	0.07 (0.26)	0.03 (0.71)	0.03 (0.67)	1				
**MID** **PARWT**	0.01 (0.83)	0.08 (0.20)	0.83 (0.00)	0.84 (0.00)	0.06 (0.36)	0.10 (0.14)	-0.00 (0.95)	0.07 (0.28)	0.07 (0.32)	-0.03 (0.66)	0.07 (0.26)	1		
**MID** **PARHT**	-0.01 (0.93)	-0.07 (0.26)	0.02 (0.72)	-0.01 (0.87)	0.08 (0.21)	0.16 (0.01)	0.32 (0.00)	-0.09 (0.19)	-0.08 (0.22)	-0.10 (0.11)	-0.02 (0.72)	0.01 (0.84)	1	
**MID** **PARBMI**	0.00 (1.00)	-0.01 (0.84)	0.02 (0.80)	0.05 (0.49)	0.12 (0.06)	0.06 (0.36)	0.01 (0.89)	1.00 (0.00)	1.00 (0.00)	-0.03 (0.66)	0.06 (0.34)	-0.07 (0.32)	0.08 (0.22)	1

Correlation

P-Value in parenthesis

Key: MATAGE- Maternal age; WTGAIN- Weight gain; PATWT-Paternal weight; MATWT-Maternal weight; PATHT-Paternal height; MATHT-Maternal height; BMI- Body mass index; PATBMI-Paternal BMI; MATBMI- Maternal BMI; BIRTHLT- Birth length; MIDPARWT-Mid-parental; MIDPARHT- Midparental height; MIDPARBMI- Mid-parental BMI.

Principal component analysis (PCA) of the studied variables gave four components. The four components explained 70.4% of the variation observed in the data (only a 29.6% loss of detail). Among the variables that loaded into the same component, the most partially correlated variable with each of the dependent variable was selected for multiple regression analysis. Maternal weight, mid-parental height, weight gain and parity were the variables with the most significant loading in their respective components and were therefore selected for multiple regression for birth length.

Results of the multiple regression analysis gave the unstandardized multiple regression model given below:

Birth Length = 38.60 + 0.39(Parity) + 0.21(Maternal weight gain) + 0.04(Maternal weight) + 0.03(Mid-parental height)

Birth Length = 38.60 + 0.39(Parity) + 0.21(Maternal weight gain) + 0.04(Maternal weight) + 0.03(Mid-parental height) …………………………………………………………………. (5)

The standardized multiple regression model is:

Birth Length = 0.20(Maternal weight gain) + 0.14(Maternal weight) + 0.13(Parity) + 0.10(Midparental height) …………………………………………………………………………………... (6)

## Discussion

The predictor variables considered in this study were mainly parental anthropometrics; they were of interest because they can be easily obtained through simple measurements. Non-anthropometric parameters included in the study are maternal age and parity; they were also considered in the study because of the ease of obtaining them along with the anthropometric parameters. While we considered information on parity reliable, we could not rely on age declared by parents with the same degree of confidence because it is generally much easier to know and remember parity than age. In many cases, the declared age was confirmed from birth certificates and hospital records. In cases where birth certificates and hospital records were not available, we relied on age declared in the questionnaire. Nevertheless, some of our subjects were either illiterates or semi-illiterates, and that increased our reservations for the correctness of the declared age. This might represent a limitation of the present study.

The summary statistics of our sample was similar to those obtained in earlier studies. In our study, the birth length of male and female babies were 48.9 cm and 49.0 cm respectively, values comparable to 51.5 cm and 50.7 cm obtained by Miletic et al.^12^ for male and female babies respectively. These values may also be compared to 50.8 cm and 50.0 cm obtained by Sajjadian et al.^13^ Maternal height of 168.1± 0.6 cm in our study and 169.7± 0.3 cm in the study of Miletic et al.^12^ were also remarkably similar. The maternal and paternal BMI of 24.3 kg/m^2^ and 25.9 kg/m^2^ respectively in our study were around the upper limit of the normal range of 18.5kg/m^2^ – 24.9kg/m^2^ according to Eknoyan^14^. In view of the implications of BMI for health, studies on distribution of BMI in Nigeria are needed to throw light on the incidence of overweight and obesity in the country.

Many of the significant simple correlations observed in this study disappeared with partial correlation analysis. This suggests that many of the studied variables were interdependent. There was a simple correlation between birth length and maternal height and between birth length and parity in our study. This was in agreement with the work of Miletic et al.^12^ These significant correlations, however, vanished with partial correlation procedure. Moreover, weight gain was a correlate of birth length in our study and those of Shapiro et al.^15^ and Lagiou et al.^16^ but the correlations reduced significantly with partial correlation analysis. These results suggested existence of complex associations between many of the studied variables. Thus, when looking at association between any two variables of complex traits, it is important to control for other variables to remove spurious correlations and prevent wrong interpretation of results.

Miletic et al.^12^ observed that maternal weight correlated with birth length and parity. Although maternal weight and parity were not significantly correlated with birth length in our study, both maternal parameters appeared in our regression model as predictors of birth length. This did not agree with the report of Knight et al.^17^ who observed that paternal height is a more important determinant of offspring birth length than maternal weight. The reasons for these discrepancies are not yet clear. Our regression model further indicated that mid-parental height was among the determinants of birth length and was a better predictor than either the paternal height or the maternal height alone. We could not ascertain whether previous workers considered mid-parental parameters in their studies; the fact that mid-parental height was a determinant of birth length in our regression model suggests an interaction between paternal and maternal height in the determination of baby's birth length. Moreover, since previous studies were carried out on different populations the discrepancies might reflect dissimilarities in genetic structure and environmental circumstances in different populations.

The regression model indicated that weight gain, maternal weight, parity, and mid-parental height are parental parameters that may be considered in determining baby's birth length. There is need for caution in applying this model because birth length is a multifactorial neonatal trait that depends on several genetic and environmental factors. This was implied by the low R-squared value obtained in the generated model. Nonetheless, the fact that the R-squared values was statistically significant suggests that the identified parental anthropometric attributes contribute to babies' birth length. Moreover, the generated model may not be applicable to other populations because of population stratification. Thus, researchers should work out possible models for predicting neonatal parameters in their respective populations. Since our model should be applied with caution, its utility therefore lies in complementing already existing conventional methods such as ultrasonography for better accuracy of baby's birth length prediction. This would encourage early intervention, and therefore prevent maternal and neonatal complications associated with abnormal birth length of babies.

## Conclusion

A model for estimating birth length of babies from easily accessible parental variables was produced. The model could complement other conventional methods of estimating baby's birth length. The derived model shows that during antenatal, focus should not be on the mother alone but also on the father as well since he also contributes to baby's birth length, an attribute of considerable obstetric importance.
